# Association of Obesity and Bariatric Surgery on Hair Health

**DOI:** 10.3390/medicina60020325

**Published:** 2024-02-14

**Authors:** Katarzyna Smolarczyk, Blazej Meczekalski, Ewa Rudnicka, Katarzyna Suchta, Anna Szeliga

**Affiliations:** 1 Department of Dermatology, Medical University of Warsaw, 02-008 Warsaw, Poland; 2 Department of Gynecological Endocrinology, Poznan University of Medical Sciences, 60-535 Poznan, Poland; blazejmeczekalski@yahoo.com (B.M.); annamaria.szeliga@gmail.com (A.S.); 3 Department of Gynecological Endocrinology, Warsaw Medical University, 00-315 Warsaw, Poland; ewa.rudnicka@poczta.onet.pl (E.R.); suchta.katarzyna@gmail.com (K.S.)

**Keywords:** obesity, hair loss, bariatric surgery

## Abstract

Obesity and obesity-related conditions today constitute a public health problem worldwide. Obesity is an “epidemic” chronic disorder, which is defined by the WHO as normal or excessive fat accumulation that may impair health. It is also defined for adults as a BMI that is greater than or equal to 30. The most common obesity-related diseases are type 2 diabetes mellitus, cardiovascular diseases, metabolic syndrome, chronic kidney disease, hyperlipidemia, hypertension, nonalcoholic fatty liver disease, and certain types of cancer. It has been also proven that obesity can have a negative effect on hair. It can lead to hair thinning. Patients with obesity can undergo bariatric surgery if they meet the inclusion criteria. The four common types of weight loss surgery include a duodenal switch with biliopancreatic diversion, laparoscopic adjustable gastric banding, Roux-en-Y gastric bypass, and sleeve gastrectomy. Bariatric surgery can affect skin and hair and is associated with telogen effluvium due to weight loss, microelement deficiency, anesthesia, low calorie intake, and low protein intake. Patients who undergo bariatric surgery can experience post-bariatric surgery depression. Hair loss can have a major impact on self-esteem, negatively affecting one’s self-image. The purpose of this narrative review is to critically review how obesity, obesity-related diseases, and bariatric surgery affect hair health in general and the hair development cycle, and how they influence hair loss.

## 1. Introduction

Obesity and metabolic syndrome, characterized by a cluster of conditions that focus on cardiovascular and diabetes-related parameters including high blood pressure, elevated blood sugar, and abnormal cholesterol levels [1], have far-reaching consequences on health. The prevalence of obesity among adults aged 20 and over in the USA (2017–2021 survey) was 41.9%, and for severe obesity, it was 9.2% [2]. It is estimated that more than 1 billion people worldwide are obese—650 million adults, 340 million adolescents, and 39 million children [3].

People who are obese can experience problems with their skin and hair [4,5]. Morianga H. et al., in their publication, indicate that obesity can accelerate hair thinning by having an impact on stem cells that are located in hair follicles [5]. It has also been described in a study by Dharam K. that a higher prevalence of MetS is seen in patients with androgenic alopecia (AA; a common cause of hair loss) when compared with that of controls. A significant association was seen also between the severity of AA and MetS [6]. 

Bariatric surgery has emerged as a viable intervention for severe obesity and metabolic syndrome, leading to substantial weight loss [7].

The four common types of weight loss surgery include a duodenal switch with biliopancreatic diversion, laparoscopic adjustable gastric banding, Roux-en-Y gastric bypass, and sleeve gastrectomy [7].

Both the American Society of Metabolic and Bariatric Surgery (ASMBS) and the International Federation for the Surgery of Obesity and Metabolic Disorders (IFSO) recommend metabolic and bariatric surgery (MBS) for individuals with a body mass index (BMI) > 35 kg/m^2^, regardless of the presence, absence, or severity of comorbidities. MBS can also be considered for individuals with metabolic disease and a BMI of 30–34.9 kg/m^2^ [7].

One of the notable repercussions of bariatric surgery on hair health is the development of telogen effluvium [8]. Telogen effluvium is a type of non-cicatricial alopecia. This condition is characterized by increased shedding and thinning of hair [9].

Post-bariatric surgery individuals commonly experience temporary hair loss, attributed to the stress of surgery, rapid weight reduction, and nutritional deficiencies [10].

Post-bariatric surgery patients often encounter nutritional deficiencies, which can further exacerbate hair-related problems. The sudden weight loss can lead to deficiencies in essential vitamins and minerals that are crucial for healthy hair, such as vitamin D, iron, zinc, and biotin [11]. It is important to address these deficiencies through targeted nutritional supplementation to mitigate post-bariatric surgery hair issues.

The purpose of this narrative review is to critically review how obesity, obesity-related diseases, and bariatric surgery affect hair health in general and the hair development cycle, and how they influence hair loss. 

Obesity-related hair loss and post-surgery hair loss can have big impact on individuals, including emotional distress which cannot be underestimated. There is a need for further exploration of obesity/bariatric surgery and hair loss.

## 2. Materials and Methods

This narrative review was conducted using a search in several major databases, including PubMed, ScienceDirect, Excerpta Medica Database, UpToDate, and Cochrane Library. The search was conducted between 1 October 2023 and 20 November 2023. Authors investigated the available data from clinical studies, review articles, and meta-analyses written in English before November 2023 that reported on hair impact, obesity, or changes in hair after bariatric surgery. Uncontrolled, nonrandomized, or case studies were not considered in our review. The following MeSH terms were used, alone or in combination: “hair”, “metabolic syndrome”, “obesity”, “bariatric surgery”, “hair loss”, “Roux-en-Y gastric bypass”, “sleeve gastrectomy”, and “telogen effluvium”. All publications were critically evaluated by the authors, including those more specifically related to the theme. Moreover, reference lists of included articles were manually screened to identify additional studies. 

## 3. Results

### 3.1. Obesity 

Obesity is regarded by many medical specialists nowadays as a worldwide epidemic [3], and the induced physiological change can influence a person’s state of health by negatively affecting various organs’ functionality [12], predisposing the individual to aging-associated diseases [13], as well as having an impact on mental health and personal well-being [14]. There are many articles describing the link between the amount of fat tissue and the physiologic changes that occur with obesity, which can have impact on skin and hair condition [5,15,16,17,18].

#### 3.1.1. Obesity and Hair Condition

Morianga H. et al., in their publication, indicate that obesity can accelerate hair thinning by having an impact on stem cells that are located in hair follicles. While during aging, an often encountered natural process is that hair follicles miniaturize, causing hair loss by the depletion of hair follicle stem cells, obesity seems to accelerate hair thinning by impacting the stem cells that are located in the hair follicles, while the physiological stress that is induced by obesity and a high-fat diet causes excessive reactive oxygen species and, as a result, epidermal keratinization of hair follicles’ stem cells, which in turns accelerates hair thinning [5]. The data presented by the authors confirm that the inflammaging of hair follicles’ stem cells can be induced by obesity and as a result can lead to organ (hair follicles) disfunction. 

Yang et al. show that a higher body mass index is significantly associated with the severity of hair loss in men with male-pattern androgenetic alopecia, and this association is even stronger among individuals with early onset of the disease [15].

Shin et al. show that obesity may be associated with premature hair graying (before the age of 30), and it is shown that the odds of experiencing premature hair graying proportionally increases with the body mass index [16].

Hair condition is also dependent on hormonal changes that may be a consequence of obesity, with some authors indicating that the scalp hair’s cortisol level among people with obesity is higher [19,20] in comparison to individuals with a lower body weight, which can cause a poorer condition and quality of hair. Papafotiou et al. indicate that obese pre-pubertal girls had higher scalp hair cortisol levels in comparison to controls [19], an observation that was also confirmed among children at the age of 6–9 by Hu et al. [19,20].

#### 3.1.2. Adipokines and Hair Condition 

Obesity is associated with adipokine changes that influence the inflammatory state of the human body [21]. Such a situation can implicate the development of several immunologically based diseases and causes aggravation of their symptoms [22,23]. One of these diseases is alopecia areata, an organ-specific autoimmune disease of the hair follicles [17]. It should be noted that the frequent coexistence of obesity and metabolic syndrome in patients with alopecia areata may indicate a common pathogenetic pathway in these conditions, hinting at an important role of the adipokines.

Serarslan G. et al., in their publication, indicate that patients with alopecia areata of the scalp have a higher serum concentration of leptin and adiponectin, and it is suggested that these adipokines may be linked to the development of the disease [12], while the secretion of leptin is dependent on the body mass index and the amount of fat tissue [12]. There are numerous studies that indicate that higher leptin levels are positively correlated with obesity-related pathologies such as metabolic syndrome, insulin resistance, cardiovascular risk, or impaired glucose tolerance [24,25,26], and some studies indicate that the pathological microcellular processes can influence hair growth and condition [27]. Thus, in this non-directive way, the leptin serum concentration may influence hair condition [12].

The adiponectin serum concentration is negatively correlated with hair loss severity, and the authors note that adiponectin may be considered a marker of hair loss severity in alopecia areata [17].

Yang et al. show that an increased serum leptin level is associated with androgenetic alopecia in men, where the levels are independent of the effects of age and obesity but positively correlated with the severity of symptoms. Still, the authors argue that an excess of plasma leptin has a negative impact on testosterone-producing Leydig cells’ function and decreases the plasma testosterone level [18], and it is suggested that the local environment of hair follicles can be affected and have an impact on the local production of testosterone and dihydrotestosterone, which has implications for the development of androgenetic alopecia [18].

#### 3.1.3. Lifestyle and Diet Influencing Hair Loss

It is widely known that most cases of obesity are caused by or linked to inappropriate diet and personal difficulties in calorie intake restriction [28,29], and that it can also be associated with specific lifestyle patterns [30,31,32,33,34]. A recent review by Minokawa [35] discussed the role of lifestyle factors that may play a role in a poorer hair condition. For example, smokers have a higher risk of developing alopecia areata in comparison to non-smokers, and it is worth noticing that cigarette smoke increases the secretion of inflammatory cytokines, as well as decreases the secretion of anti-inflammatory cytokines. Moreover, sleep disorders may be involved in a higher risk of alopecia, such as insomnia or a sleep apnea syndrome, which is more frequent among people with obesity. In relation to patients with alopecia areata, it was suggested that sleep disturbances may lead to Th1 immune responses.

Among people with obesity, a lower serum concentration of vitamin D is widely observed [36,37,38], and it is known that vitamin D plays a role in immunomodulation and has an anti-inflammatory effect [39]. Almohanna et al. [19] suggest that vitamin D modulates the growth and differentiation of keratinocytes through binding to the nuclear vitamin D receptor and thus influences the hair condition [39].

Excessive intake of gluten, as a cause of inappropriate diet, was mentioned as another factor that can negatively influence the condition of hair: gluten enhances Th17-mediated skin inflammation and can exacerbate hair follicle inflammation. A strikingly excessive intake of omega-6 fatty acids may contribute to the development of inflammatory skin diseases by elevation of the serum concentration of inflammatory lipid mediators—leukotriene B4 and prostaglandin E2 [35].

Among people with obesity, a lower serum concentration of vitamin D is widely observed. Almohanna HM. et al., in their analysis, indicated that vitamin D plays a role in immunomodulation and has an anti-inflammatory effect [35]. Vitamin D modulates the growth and differentiation of keratinocytes through binding to the nuclear vitamin D receptor and influences hair condition.

Obesity is a multifaceted disease that impacts many organs and involves cellular, hormonal, and cytokine changes that can influence skin and hair condition. An inappropriate diet that is rich in omega-6 fatty acids and poor in vitamins, which is commonly observed among obese people, aggravates this condition. In addition, obesity-dependent comorbidities such as sleep disturbances, but also metabolic consequences, have a negative impact on skin appendages [39]. 

### 3.2. Metabolic Syndrome 

#### 3.2.1. Diabetes Mellitus and Hair Loss

Diabetes mellitus is a metabolic disorder that is associated with abnormally high blood glucose levels (hyperglycemia) that adversely affects many organs, leading to various complications, including retinopathy, nephropathy, and neuropathy [40,41], through the modulation of the Wnt/ß-catenin signaling pathway [40,41]. In a recent study by Wang M et al., it was shown that diabetes can inhibit hair regrowth by inhibiting Wnt/ß-catenin signaling [42]. The activation of Wnt/ß-catenin promotes the growth, morphogenesis, and regeneration of hair follicles, but when the cells are not stimulated by Wnt ligands, ß-catenin is phosphorylated by the destruction complex and subsequently ubiquitinated and degraded [42]. An animal model showed that the depilation-induced anagen phase was delayed in db/db mice, high-fat diet (HFD), and streptozotocin (STZ)-induced diabetic mice [43]. But in diabetic mice, hair regrowth and wound-induced hair follicle neogenesis (WIHN) were reduced because of the suppression of Wnt/ß-catenin signaling and decreased proliferation of hair follicle cells [43].

Many other authors have previously confirmed the relationship between hair loss and diabetes [44,45]. In a cross-sectional study of 326 women, the prevalence of type 2 diabetes was higher among women with severe central hair loss (17.6%) than among those without severe hair loss (5.7%) [44]. Androgenetic alopecia (AA), the most common form of hair thinning in non-Black populations, has also been associated with insulin resistance (IR) and other manifestations of the metabolic syndrome [45,46]. AA stems from the effects of the testosterone metabolite dihydrotestosterone on androgen-sensitive hair follicles, suggesting that vascular impairment due to hyperglycemia could damage hair follicles and contribute to hair loss [44]. Vasoactive substances that are released due to endothelial dysfunction in insulin resistance cause perifollicular vasoconstriction and proliferation in smooth muscle cells in the vascular wall [42]. As a result, microvascular circulation is impaired, local tissue hypoxia occurs, and a progressive miniaturization is observed in their follicles [18]. The association between IR and hyperinsulinemia has been reported in many studies [47,48]. In a study by Swaroop et al., men with AA were found to have higher fasting insulin levels than those in the control group [45], while another study showed a significant relationship between IR and early-onset AA [47,48,49].

#### 3.2.2. Hypertension and Hair Loss

The association between alopecia and coronary heart disease and hypertension has also been investigated for over 70 years [50]. Although some studies have found no relationship, most of them supported the dependence [51]. 

Specifically, studies have shown that coronary heart disease is more likely to occur in individuals with alopecia compared to non-bald individuals, and in the group with alopecia, higher cholesterol levels and higher blood pressure were found [51]. In a study by Arias-Santiago S. et al., the mean systolic and diastolic blood pressure were significantly higher in women with AA than in the control group [52]. They also observed that the mean aldosterone levels were significantly higher in the AA patient group, suggesting that elevated aldosterone values in these patients may contribute, alongside other mechanisms, to the development of androgenetic alopecia and may also explain the higher prevalence of hypertension. These authors proposed blood pressure screening of women with AA to achieve earlier diagnosis of an unsuspected hypertension and to introduce appropriate treatment. The elevated blood pressure in patients with AA might also be explained by an increase in peripheral vascular sensitivity to androgens. The androgens that are involved in the AA pathogenesis bind to blood vessel receptors, increasing the likelihood of a blood pressure increase [51,52]

#### 3.2.3. Lipid Profile and Hair Loss

One of the components of the metabolic syndrome is dyslipidemia. Many studies have been published examining the relationship between AA and dyslipidemia [52,53,54]. A meta-analysis by Kim et al. analyzed 19 studies related to dyslipidemia and AA and concluded that serum total of cholesterol, triglyceride, and low-density lipoprotein cholesterol levels in the AA group were higher, and high-density lipoprotein cholesterol values were lower compared to the control group [54]. The results are explained by an increased sensitivity to androgens in AA patients, with an underlying relationship between AA, dyslipidemia, and chronic microinflammation. Similar results were previously obtained [44,53,55], indicating that proinflammatory cytokines lead to disturbances in the serum lipid profile through changes in cholesterol transport and apolipoproteins [54]. A meta-analysis regarding alopecia and its association with coronary heart disease and cardiovascular risk factors also found a positive correlation between alopecia and an elevated serum concentration of cholesterol and triglyceride [51].

In conclusion, alopecia shows a well-established relationship with cardiovascular risks, including insulin resistance, hyperlipidemia, and hypertension, which establishes a strong correlation of hair cell loss with metabolic syndrome.

### 3.3. Bariatric Surgery

#### 3.3.1. Post-Surgery Tellogen Effluvium

Bariatric surgery (BS), known as weight loss surgery, has four commonly implemented types: a duodenal switch with biliopancreatic diversion; laparoscopic adjustable gastric banding; Roux-en-Y gastric bypass; and sleeve gastrectomy. In general, the surgery affects hormonal balance and hair and skin condition [7]. Usually, within the first three months after bariatric surgery, telogen effluvium, a type of a non-cicatricial alopecia that is characterized by increased hair shedding and thinning of hair, occurs abruptly [56]. The mechanisms of telogen effluvium include premature anagen phase interruption, excessive prolongation of the anagen phase, reduced anagen phase duration, and delayed teleptosis [9]. The main causes of telogen effluvium include [9,57] physiologic factors, infection, endocrine factors, organ dysfunction, stress, local causes, nutritional factors, and diseases like systemic lupus erythematosus or ones of an idiopathic nature, as shown in Table 1.

Telogen effluvium usually occurs abruptly within the first three months after bariatric surgery. The diagnosis of telogen effluvium can be made by taking medical history, performing a pull test, trichogram, trichoscopy, and interpreting laboratory findings. Other methods include hair weighing, a wash test, unit area trichogram, and trichoscan (for further information, see Appendix A).

According to a meta-analysis which included 18 studies on hair loss after MBS, the incidence is more common in women, and patients with hair loss are younger than controls. The overall incidence of hair loss ranges from 4.5% to 80%. The pooled incidence of hair loss was established to be 57% (95%CI, 42–71%), and it decreases with longer follow-up times [10]. In most cases, telogen effluvium is a self-limiting condition, but it can cause a trauma. Therefore, treatment with minoxidil and topical steroids is implemented [9].

#### 3.3.2. Post-Surgery Nutritional Deficiencies

The number of studies evaluating the link between nutritional deficiencies after bariatric surgery (BS), which negatively influences hair status, is limited.

According to data from the literature, different types of bariatric surgeries have a different influence on micronutrient absorption. Such procedures, like laparoscopic adjustable gastric banding (LAGB) and sleeve gastrectomy (SG), have an impact on iron, zinc, selenium, folate, and Vitamin B12 absorption [58]. Procedures like Roux-en-Y gastric bypass (RYGB) and jejunoileal bypass (JIB) have a more profound influence on the absorption of essentials vitamins, minerals, and trace elements [58].

Katsogridaki et al. evaluated the prevalence of hair loss after laparoscopic sleeve gastrectomy (LSG) [11]. The authors evaluated serum parameters such as iron, zinc, folic acid, vitamin B12, total protein, and albumin before bariatric surgery and 6 months after surgery. The prevalence of hair loss was 56% after LSG, while hair loss was related to a decrease in serum zinc, iron, and vitamin B12. According to this study, preoperative monitoring and counseling of these macronutrients may be a preventive and therapeutic measure.

Ruis-Tovar et al. studied 42 obese patients before and after laparoscopic sleeve gastrectomy (LSG) and monitored the incidence of hair loss, while micronutrients were evaluated before surgery and 3, 6, and 12 months after surgery [59]. A significant association was observed between hair loss and zinc and iron levels, eventually leading to the conclusion that zinc and iron can be a good predictor of hair loss [41]. Rojas P et al. evaluated the nutritional status of zinc, iron, cooper, selenium, and visceral protein in women with different degrees of hair loss after gastric bypass or tubular gastrectomy, from which it was concluded that a higher zinc and iron intake has a protective effect on hair loss at six months after surgery [60].

According to Ladoux’s study, after bariatric surgery, the most frequent nutritional symptom is hair loss, which is mainly associated with iron and protein deficiency, and thus, iron and protein supplementation after BS can help avoid hair loss [61]. Similar suggestions are provided by Gasmi et al. [62], who suggest that a proper diet containing sufficient nutrients and multivitamin supplements can help ameliorate the health consequences of micronutrient deficiency.

#### 3.3.3. Influence of Body Weight Loss after BS on Hair

The body weight loss following bariatric surgery is associated with subsequent hair loss [61]. A large proportion of patients complain of hair loss in the immediate time after surgery. This symptom is less frequent in the later stages (*p* < 0.001) [8]. More specifically, 79 percent of 315 patients reported hair loss between the third- and fourth-month interval, which continued for an average of 5.5 ± 2.6 months, while permanent alopecia was not observed in any of the patients [8]. The onset time and end time of hair loss in one study was 3.4 ± 1.4 and 9.03 ± 3.6 months, respectively [63]. A retrospective study conducted by Ledoux et al. found that the incidence of hair loss decreases from 65% at 12 months to 35% at 12 months follow-up. Most of the weight loss after bariatric surgery occurred in the first year after surgery, and with this period being characterized by the fastest weight loss. Typically, the faster the body mass loss was, the more intense hair loss was observed [61].

No difference in hair loss was observed in terms of the type of bariatric surgery; for example, there was no difference between Roux-en-Y gastric bypass and sleeve gastrectomy [61]. There was also no difference in the intensity of hair loss when comparing patients after laparoscopic gastric application and laparoscopic sleeve gastrectomy [64], but the intensity of the symptom depends also on the degree of weight loss. It was noticed that the postoperative weight was lower in subjects with hair loss than in those without, both in the short term and long term, and both caloric and protein intakes were lower in the short term [62]. A significantly lower weight was observed in patients claiming hair loss compared to patients without this symptom both in the short term, as well as in the long term (*p* < 0.001). Nevertheless, there was no such statistical difference according to the percentage of weight loss.

Although published information on hair loss after gastric bypass is limited, one study reported that 56% of patients experienced hair loss despite taking a daily multivitamin [60]. Those observations were confirmed in another study, where of 42 patients, 15 took oral medication (6 with ferrous sulfate, 5 with decavitamin, and 4 with zinc gluconate oral solution) against hair loss, with no obvious improvement. In contrast, in another study, postoperative supplements significantly decreased hair loss, and patients who were prescribed supplementation claimed hair loss in 40% of cases, whereas those without supplementation presented the symptom in 60% of cases (*p* = 0.045). Those observations may indicate that micronutrient deficiencies are important in the pathomechanism of hair loss, although, most likely, they are not the only reason. It is possible that the loss of subcutaneous tissue makes it difficult for the scalp to support hair. It has been proposed that women have longer hair than men and have higher requirements for scalp support [61].

A possible mechanism that is responsible for hair loss associated with bariatric surgery is physical stress due to rapid weight loss [63]. A decreasing body weight after surgery causes more hair to enter the resting phase, and a resting phase can last for 3+ months on average. At the end of this phase, major hair loss might occur, usually at 3–6 months after surgery [10].

## 4. Conclusions

In essence, this narrative review aims to elevate the understanding of the complex relationship between obesity, its treatment, and hair health. We advocate for the need to address the often-neglected psychological and aesthetic concerns of obese individuals seeking bariatric surgery.

While often overlooked, hair thinning and alopecia become pertinent issues in the context of obesity treatment, primarily because many obese individuals seek bariatric surgery for improvements in physical appearance and self-esteem, aspects that are closely tied to hair health. 

There is also a lack of comparative studies examining the improvements in hair health following anti-obesity treatments in obese subjects. This gap in research highlights the need for more rigorous, well-designed studies that focus on understanding and preventing hair loss post bariatric surgery. 

Furthermore, exploring the potential of hair health as a marker of the global metabolic state in obese patients, both pre- and post-BS, could offer valuable insights into the systemic effects of obesity and its treatments. 

## Figures and Tables

**Table 1:** Factors inducing telogen effluvium [9,57].

Factors	Selected Causes
physiological	Postpartum
	Shedding of the new-born hair
	Seasonal shedding
infection	COVID-19
	Syphilis
	HIV infection
	Malaria
endocrine	Hyperthyroidism
	Hypothyroidism
	Hormonally active tumors
	of the ovaries, pituitary, and adrenal glands
organ dysfunction	Renal failure
	Hepatic failure
stress	Surgery
	Psychological stress
local causes	Hair transplant
	Inflammatory disorders of the scalp
Nutritional factors	Crash diets
	Iron deficiency
	Zinc deficiency
	Vitamin B2 or B 12 deficiency
	Vitamin D3 deficiency
drugs	Antithrombotic drugs: heparin, heparin derivatives, coumarin
	Cardiology drugs including β-blockers (β-adrenolytics), Angiotensin inhibitors, calcium channel blockers.
	Hypolipidaemic drugs: fibrates, butyrophenone
	Hormones: androgens, danazole, oral contraceptives
other	Systemic lupus erythematosus
	Dermatomyositis
	Systemic sclerosis
	Keratolytics shampoo
	Idiopathic causes

## Abbreviations

AA (androgenetic alopecia); ASMBS (American Society of Metabolic and Bariatric Surgery); aTg (thyroglobulin antibodies); aTPO (thyroid peroxidase antibodies); BMI (body mass index); BS (bariatric surgery); CKD (chronic kidney disease); CRP (C-reactive protein); CVDs (cardiovascular diseases); HFD (high-fat diet); IFSO (International Federation for the Surgery of Obesity and Metabolic Disorders); IR (insulin resistance); JIB (jejunoileal bypass); LAGB (laparoscopic adjustable gastric banding); LSG (laparoscopic sleeve gastrectomy); MetS (metabolic syndrome); NAFLD (nonalcoholic fatty liver disease); RYGB (gastric bypass); SG (sleeve gastrectomy); STZ (streptozotocin); T2DM (type 2 diabetes mellitus); TSH (thyroid-stimulating hormone); WHO (World Health Organization); WIHN (wound-induced hair follicle neogenesis).

## Appendix A

### Hair Health Diagnostics

The diagnosis of telogen effluvium can be made by taking medical history, performing a pull test, trichogram, trichoscopy, and interpreting laboratory findings. Other methods include hair weighing, a wash test, unit area trichogram, and trichoscan.

The pull test, also known as the “traction test” is a diagnostic tool which can be useful in a diagnosis of excessive shedding. It helps measure the severity and location of hair loss [65]. The pull test is positive when the physician pulls 40–60 hairs, and 10% of the hair (5–6 hairs) falls out (with no hair washing for 5 days. Some criteria state that when more than 2 hairs fall out, the test can be considered positive) [66].

Trichogram, also called the pluck test, can be a useful diagnostic semi-invasive method. Hairs are taken from specified sites at least 2(5) days after the last shampooing. To start, 60–80 hairs are grasped with a hemostat that is covered with rubber and taken foro the microscope analysis [20]. The normal trichogram values are 66–96% anagen hairs, 0–6% catagen hairs, 2–18% telogen hairs, and 0–18% dysplastic/dystrophic hairs. In telogen effluvium, the percentage of telogen hairs equals 20–25% [67].

Trichoscopy is a non-invasive diagnostic method which includes the usage of a dermoscope/trichoscope. Trichoscopy findings include mild perifollicular scaling around terminal hairs and an absence of follicular orifices (dots) in areas with hair loss. The loss of eyebrows and perifollicular facial papules may also occur [67].

Laboratory findings are of use while making the diagnosis, including complete blood count with differential, ferritin, iron, TSH (thyroid-stimulating hormone), aTg, (thyroglobulin antibodies), aTPO, (thyroid peroxidase antibodies), CRP (C-reactive protein), prolactin, fasting glucose, total protein, and progesterone [57]. Other blood tests may be indicated, depending on the patient’s history.

We propose that future research should delve deeper into the etiological factors—lifestyle, nutritional, hormonal, and associated comorbidities—that influence hair loss in obese individuals undergoing BS. Such studies could illuminate the broader implications of obesity and its treatment on individuals’ overall health and well-being.

## References

[1] Yang M., Liu S., Zhang C. (2022). The Related Metabolic Diseases and Treatments of Obesity. Healthcare.

[2] Stierman B., Afful J., Carroll M.D., Chen T.-C., Davy O., Fink S., Fryar C.D., Gu Q., Hales C.M., Hughes J.P. (2021). National health and nutrition examination survey 2017–March 2020 prepandemic data files-development of files and prevalence estimates for selected health outcomes. Natl. Health Stat. Rep..

[3] WHO (2021). Obesity and Overweight—Fact Sheet. www.who.int/news-room/fact-sheets/detail/obesity-and-overweight.

[4] Darlenski R., Mihaylova V., Handjieva-Darlenska T. (2022). The Link Between Obesity and the Skin. Front. Nutr..

[5] Morinaga H., Mohri Y., Grachtchouk M., Asakawa K., Matsumura H., Oshima M., Takayama N., Kato T., Nishimori Y., Sorimachi Y. (2021). Obesity accelerates hair thinning by stem cell-centric converging mechanisms. Nature.

[6] Dharam Kumar K., Kishan Kumar Y., Neladimmanahally V. (2018). Association of androgenetic alopecia with metabolic syndrome: A case–control study on 100 patients in a tertiary care hospital in South India. Indian J. Endocrinol. Metab..

[7] Eisenberg D., Shikora S.A., Aarts E., Aminian A., Angrisani L., Cohen R.V., De Luca M., Faria S.L., Goodpaster K.P.S., Haddad A. (2022). 2022 American Society for Metabolic and Bariatric Surgery (ASMBS) and International Federation for the Surgery of Obesity and Metabolic Disorders (IFSO): Indications for Metabolic and Bariatric Surgery. Surg. Obes. Relat. Dis..

[8] Şen O., Türkçapar A.G. (2021). Hair Loss After Sleeve Gastrectomy and Effect of Biotin Supplements. J. Laparoendosc. Adv. Surg. Tech..

[9] Asghar F., Shamim N., Farooque U., Sheikh H., Aqeel R. (2020). Telogen Effluvium: A Review of the Literature. Cureus.

[10] Zhang W., Fan M., Wang C., Mahawar K., Parmar C., Chen W., Yang W., Global Bariatric Research Collaborative (2021). Hair Loss After Metabolic and Bariatric Surgery: A Systematic Review and Meta-analysis. Obes. Surg..

[11] Katsogridaki G., Tzovaras G., Sioka E., Perivoliotis K., Zachari E., Magouliotis D., Tasiopoulou V., Chatedaki C., Zacharoulis D. (2018). Hair Loss After Laparoscopic Sleeve Gastrectomy. Obes. Surg..

[12] Serarslan G., Özcan O., Okyay E., Ünlü B., Karadağ M. (2020). Role of adiponectin and leptin in patients with alopecia areata with scalp hair loss. Ir. J. Med. Sci..

[13] Fang P., She Y., Yu M., Min W., Shang W., Zhang Z. (2023). Adipose–Muscle crosstalk in age-related metabolic disorders: The emerging roles of adipo-myokines. Ageing Res. Rev..

[14] Avila C., Holloway A.C., Hahn M.K., Morrison K.M., Restivo M., Anglin R., Taylor V.H. (2015). An Overview of Links Between Obesity and Mental Health. Curr. Obes. Rep..

[15] Yang C.C., Hsieh F.N., Lin L.Y., Hsu C.K., Sheu H.M., Chen W. (2014). Higher body mass index is associated with greater severity of alopecia in men with male-pattern androgenetic alopecia in Taiwan: A cross-sectional study. J. Am. Acad. Dermatol..

[16] Shin H., Ryu H.H., Yoon J., Jo S., Jang S., Choi M., Kwon O., Jo S.J. (2015). Association of premature hair graying with family history, smoking, and obesity: A cross-sectional study. J. Am. Acad. Dermatol..

[17] Stochmal A., Waśkiel-Burnat A., Chrostowska S., Zaremba M., Rakowska A., Czuwara J., Rudnicka L. (2021). Adiponectin as a novel biomarker of disease severity in alopecia areata. Sci. Rep..

[18] Yang C., Chung P., Lin L., Hughes M.W., Tsai Y. (2017). Higher plasma leptin is associated with higher risk of androgenetic alopecia in men. Exp. Dermatol..

[19] Papafotiou C., Christaki E., van den Akker E.L.T., Wester V.L., Apostolakou F., Papassotiriou I., Chrousos G.P., Pervanidou P. (2017). Hair cortisol concentrations exhibit a positive association with salivary cortisol profiles and are increased in obese prepubertal girls. Stress.

[20] Hu J.J., Duan X.N., Fang J., Xu N., Wan Y.H., Su P.Y., Tao F.B., Sun Y. (2017). Association between hair cortisol concentration and overweight and obesity in 6–9 years old childhood. Zhonghua Yu Fang Yi Xue Za Zhi.

[21] Nakamura K., Fuster J.J., Walsh K. (2013). Adipokines: A link between obesity and cardiovascular disease. J. Cardiol..

[22] Schulze P.C., Biolo A., Gopal D., Shahzad K., Balog J., Fish M., Siwik D., Colucci W.S. (2011). Dynamics in Insulin Resistance and Plasma Levels of Adipokines in Patients With Acute Decompensated and Chronic Stable Heart Failure. J. Card. Fail..

[23] Fujimaki S., Kanda T., Fujita K., Tamura J., Kobayashi I. (2001). The Significance of Measuring Plasma Leptin in Acute Myocardial Infarction. J. Int. Med. Res..

[24] Ghantous C.M., Azrak Z., Hanache S., Abou-Kheir W., Zeidan A. (2015). Differential Role of Leptin and Adiponectin in Cardiovascular System. Int. J. Endocrinol..

[25] Yun J.E., Kimm H., Jo J., Jee S.H. (2010). Serum leptin is associated with metabolic syndrome in obese and nonobese Korean populations. Metabolism.

[26] Lee S.W., Jo H.H., Kim M.R., You Y.O., Kim J.H. (2011). Association between metabolic syndrome and serum leptin levels in postmenopausal women. J. Obstet. Gynaecol..

[27] Dopytalska K., Baranowska-Bik A., Roszkiewicz M., Bik W., Walecka I. (2020). The role of leptin in selected skin diseases. Lipids Health Dis..

[28] Safaei M., Sundararajan E.A., Driss M., Boulila W., Shapi’I A. (2021). A systematic literature review on obesity: Understanding the causes & consequences of obesity and reviewing various machine learning approaches used to predict obesity. Comput. Biol. Med..

[29] Ludwig D.S., Ebbeling C.B. (2018). The Carbohydrate-Insulin Model of Obesity: Beyond “Calories in, Calories Out”. JAMA Intern. Med..

[30] Verduci E., Bronsky J., Embleton N., Gerasimidis K., Indrio F., Köglmeier J., de Koning B., Lapillonne A., Moltu S.J., Norsa L. (2021). Role of Dietary Factors, Food Habits, and Lifestyle in Childhood Obesity Development: A Position Paper From the European Society for Paediatric Gastroenterology, Hepatology and Nutrition Committee on Nutrition. J. Pediatr. Gastroenterol. Nutr..

[31] Aljefree N.M., Shatwan I.M., Almoraie N.M. (2022). Impact of the Intake of Snacks and Lifestyle Behaviors on Obesity among University Students Living in Jeddah, Saudi Arabia. Healthcare.

[32] Pereira L.J., Hinnig P.d.F., Matsuo L.H., Di Pietro P.F., de Assis M.A.A., Vieira F.G.K. (2022). Association between lifestyle patterns and overweight and obesity in adolescents: A systematic review. Br. J. Nutr..

[33] Lonnie M., Wadolowska L., Morze J., Bandurska-Stankiewicz E. (2022). Associations of Dietary-Lifestyle Patterns with Obesity and Metabolic Health: Two-Year Changes in MeDiSH^®^ Study Cohort. Int. J. Environ. Res. Public Health.

[34] Ryan D.H. (2023). Lifestyle-Based Obesity Care. Gastroenterol. Clin. North Am..

[35] Minokawa Y., Sawada Y., Nakamura M. (2022). Lifestyle Factors Involved in the Pathogenesis of Alopecia Areata. Int. J. Mol. Sci..

[36] Bennour I., Haroun N., Sicard F., Mounien L., Landrier J.-F. (2022). Vitamin D and Obesity/Adiposity—A Brief Overview of Recent Studies. Nutrients.

[37] Vranić L., Mikolašević I., Milić S. (2019). Vitamin D deficiency: Consequence or cause of obesity?. Medicina.

[38] Pereira-Santos M., Costa P.R.F., Assis A.M.O., Santos C.A.S.T., Santos D.B. (2015). Obesity and vitamin D deficiency: A systematic review and meta-analysis. Obes. Rev..

[39] Almohanna H.M., Ahmed A.A., Tsatalis J.P., Tosti A. (2018). The Role of Vitamins and Minerals in Hair Loss: A Review. Dermatol. Ther..

[40] Chen Q., Ma J.-X. (2017). Canonical Wnt signaling in diabetic retinopathy. Vis. Res..

[41] Qi W., Yang C., Dai Z., Che D., Feng J., Mao Y., Cheng R., Wang Z., He X., Zhou T. (2014). High Levels of Pigment Epithelium–Derived Factor in Diabetes Impair Wound Healing Through Suppression of Wnt Signaling. Diabetes.

[42] Wang M., Yao S., He D., Qahar M., He J., Yin M., Wu J., Yang G. (2022). Type 2 Diabetic Mellitus Inhibits Skin Renewal through Inhibiting WNT-Dependent Lgr5+ Hair Follicle Stem Cell Activation in C57BL/6 Mice. J. Diabetes Res..

[43] Kyei A., Bergfeld W.F., Piliang M., Summers P. (2011). Medical and environmental risk factors for the development of central centrifugal cicatricial alopecia: A population study. Arch. Dermatol..

[44] El Sayed M.H., Abdallah M.A., Aly D.G., Khater N.H. (2016). Association of metabolic syndrome with female pattern hair loss in women: A case-control study. Int. J. Dermatol..

[45] Su L.-H., Chen T.-H. (2010). Association of androgenetic alopecia with metabolic syndrome in men: A community-based survey. Br. J. Dermatol..

[46] Sharma L., Dubey A., Gupta P., Agrawal A. (2013). Androgenetic alopecia and risk of coronary artery disease. Indian Dermatol. Online J..

[47] Swaroop M.R., Kumar B.M., Sathyanarayana B., Yogesh D., Raghavendra J., Kumari P. (2019). The association of metabolic syndrome and insulin resistance in early-onset androgenetic alopecia in males: A case–control study. Indian J. Dermatol..

[48] Acibucu F., Kayatas M., Candan F. (2010). The association of insulin resistance and metabolic syndrome in early androgenetic alopecia. Singapore Med. J..

[49] Pengsalae N., Tanglertsampan C., Phichawong T., Lee S. (2013). Association of early-onset androgenetic alopecia and metabolic syndrome in Thai men: A case-control study. J. Med. Assoc. Thai..

[50] Friedlich A.L. (1955). Book Reviews: Coronary Heart Disease in Young Adults: A Multidisciplinary Study. By Menard M. Gertler, M. D., and Paul D. White, M. D.: With aid, advice, and editorial assistance of E. F. Bland, M. D., and others. Cloth. $5. Pp. 218, with illustrations. Published for Commonwealth Fund by Harvard University Press, Cambridge 38, Mass.; Oxford University Press, Amen House, Warwick Square, London, E. C. 4, England, 1954. Angiology.

[51] Trieu N., Eslick G.D., Trieu N.E. (2014). Alopecia and its association with coronary heart disease and cardiovascular risk factors: A meat analysis. Int. J. Cardiol..

[52] Arias-Santiago S., Gutiérrez-Salmerón M., Buendía-Eisman A., Girón-Prieto M., Naranjo-Sintes R. (2009). Hypertension and aldosterone levels in women with early-onset androgenetic alopecia. Br. J. Dermatol..

[53] Sadighha A., Zahed G. (2008). Evaluation of lipid levels in androgenetic alopecia in comparison with control group. J. Eur. Acad. Dermatol. Venereol..

[54] Kim M., Shin I., Yoon H., Cho S., Park H. (2016). Lipid profile in patients with androgenetic alopecia: A meta-analysis. J. Eur. Acad. Dermatol. Venereol..

[55] Chakrabarty S., Hariharan R., Gowda D., Suresh H. (2014). Association of premature androgenetic alopecia and metabolic syndrome in a young Indian population. Int. J. Trichology.

[56] A Cohen-Kurzrock R., Cohen P.R. (2021). Bariatric Surgery-Induced Telogen Effluvium (Bar SITE): Case Report and a Review of Hair Loss Following Weight Loss Surgery. Cureus.

[57] Brzezińska-Wcisło L., Rakowska A., Rudnicka L., Bergler-Czop B., Czuwara J., Maj J., Olszewska M., Placek W., Reich A. (2018). Łysienie androgenowe kobiet i mężczyzn. Rekomendacje diagnostyczno-terapeutyczne Polskiego Towarzystwa Dermatologicznego. Dermatol. Rev./Przegląd Dermatol..

[58] Mechanick J.I., Apovian C., Brethauer S., Garvey W.T., Joffe A.M., Kim J., Kushner R.F., Lindquist R., Pessah-Pollack R., Seger J. (2019). Clinical practice guidelines for the perioperative nutrition, metabolic, and nonsurgical support of patients undergoing bariatric procedures—2019 Update. Endocr. Pract..

[59] Ruiz-Tovar J., Oller I., Llavero C., Zubiaga L., Diez M., Arroyo A., Calero A., Calpena R. (2014). Hair Loss in Females after Sleeve Gastrectomy: Predictive Value of Serum Zinc and Iron Levels. Am. Surg..

[60] Rojas P., Gosch M., Basfi-Fer K., Carrasco F., Codoceo J., Inostroza J., Valencia A., Adjemian D., Rojas J., Díaz E. (2012). Alopecia in women with severe and morbid obesity who undergo bariatric surgery. Nutr. Hosp..

[61] Ledoux S., Flamant M., Calabrese D., Bogard C., Sami O., Coupaye M. (2020). What Are the Micronutrient Deficiencies Responsible for the Most Common Nutritional Symptoms After Bariatric Surgery?. Obes. Surg..

[62] Gasmi A., Bjørklund G., Mujawdiya P.K., Semenova Y., Peana M., Dosa A., Piscopo S., Benahmed A.G., Costea D.O. (2021). Micronutrients deficiences in patients after bariatric surgery. Eur. J. Nutr..

[63] Guo H., Zhu J., Ma Y., Sachin B., Cao D., Tang L. (2017). Risk factors analysis of hair loss in obese patients afer laparoscopic sleeve gastrectomy. Chin. J. Dig. Surg..

[64] Wang C., Yang C., Yang J. (2014). Surgical results of laparoscopic Roux-en-Y gastric bypass in super obese patients with BMI (greater-than or equal to) 60 in China. Surg. Laparosc. Endosc. Percutan Tech..

[65] Dhurat R., Saraogi P. (2009). Hair evaluation methods: Merits and demerits. Int. J. Trichology.

[66] McDonald K.A., Shelley A.J., Colantonio S., Beecker J. (2017). Hair pull test: Evidence-based update and revision of guidelines. J. Am. Acad. Dermatol..

[67] Olszewska M., Warszawik O., Rakowska A., Słowińska M., Rudnicka L. (2011). Methods of hair loss evaluation in patients with endocrine disorders. Endokrynol. Pol..

